# Do MicroRNAs Modulate Visceral Pain?

**DOI:** 10.1155/2018/5406973

**Published:** 2018-10-10

**Authors:** Zhuo-Ying Tao, Yang Xue, Jin-Feng Li, Richard J. Traub, Dong-Yuan Cao

**Affiliations:** ^1^Key Laboratory of Shaanxi Province for Craniofacial Precision Medicine Research, Research Center of Stomatology, Xi'an Jiaotong University College of Stomatology, 98 West 5th Road, Xi'an, Shaanxi 710004, China; ^2^Department of Oral and Maxillofacial Surgery, Xi'an Jiaotong University College of Stomatology, 98 West 5th Road, Xi'an, Shaanxi 710004, China; ^3^Department of Neural and Pain Sciences, School of Dentistry, Center to Advance Chronic Pain Research, University of Maryland, 650 West Baltimore Street, Baltimore, Maryland 21201, USA

## Abstract

Visceral pain, a common characteristic of multiple diseases relative to viscera, impacts millions of people worldwide. Although hundreds of studies have explored mechanisms underlying visceral pain, it is still poorly managed. Over the past decade, strong evidence emerged suggesting that microRNAs (miRNAs) play a significant role in visceral nociception through altering neurotransmitters, receptors and other genes at the posttranscriptional level. Under pathological conditions, one kind of miRNA may have several target mRNAs and several kinds of miRNAs may act on one target, suggesting complex interactions and mechanisms between miRNAs and target genes lead to pathological states. In this review we report on recent progress in examining miRNAs responsible for visceral sensitization and provide miRNA-based therapeutic targets for the management of visceral pain.

## 1. Introduction

Epigenetic modifications including DNA methylation, histone modifications, and noncoding RNAs can enhance or suppress gene expression pre- or post-transcriptionally without changing the primary DNA sequence [1–3]. Among noncoding RNAs, microRNA (miRNAs) are well studied and verified as contributing to pathological states, including cancer, cardiovascular, neurodegenerative, and autoimmune disease [4–7]. A 2007 study identified the involvement of miRNAs in the development and maintenance of orofacial inflammatory pain becoming the first report to explore the role of miRNAs in pain [8]. Over the subsequent decade, selective miRNAs acting on different target genes have been investigated in various pain disorders in both human conditions and animal models. The goal of this review is to update current knowledge on miRNAs in visceral pain and discuss the mechanisms involved, providing new potential targets for visceral pain management clinically.

## 2. Characteristics of Visceral Pain

Visceral pain, defined as pain originating from the internal organs, is a hallmark feature of multiple diseases and disorders including visceral inflammation (e.g., inflammatory bowel disease, pancreatitis, and appendicitis), occlusion of bile or urine flow (e.g., gallstones, kidney stones), reflux of stomach acid (e.g., gastroesophageal reflux disease), functional visceral disorders (e.g., irritable bowel syndrome (IBS), endometriosis, interstitial cystitis/bladder pain syndrome (IC/BPS), and functional dyspepsia), and ischemia (e.g., angina, colic) [9–16]. Visceral pain affects approximately 25% of people worldwide and is poorly managed clinically because it is typically defined as a symptom of another condition and not a disease itself [17].

Understanding the characteristics and pain pathways is useful to appreciate visceral pain modulation and potential therapeutics. Cervero and Laird first mentioned clinical features of visceral pain that discriminate it from somatic pain [18]. Visceral pain is not evoked from all viscera and may not be associated with tissue injury. Visceral pain can be produced by distending hollow organs, ischemia, or inflammation, but not stimuli which can induce somatic pain like crushing, cutting, or burning [19]. These noxious stimuli activate spinally projecting visceral afferent fibers (A*δ*- and C-fibers) which terminate throughout the dorsoventral and mediolateral extent of the dorsal horn. Second-order neurons transmit the nociceptive information to the brain through multiple pathways including the spinothalamic, spinoreticular, spinoparabrachial tracts, and the postsynaptic dorsal columns-medial lemniscal pathway [20–22]. Tertiary neurons in the thalamus and brainstem send ascending projections to different regions of the cortex and diencephalon which manage different aspects of visceral nociception. The primary and secondary somatosensory cortices confer sensory-discriminative aspects of pain perception (e.g., location, duration, quality, and intensity). Limbic structures (amygdala, insula, anterior and midcingulate cortex, and hypothalamus) manage affective and motivational aspects of pain. Compared with somatic pain, visceral pain is diffuse and hard to accurately localize. It refers to other locations on the body because the afferent fibers from one viscera may converge in the spinal cord with fibers from other visceral organs and nonvisceral tissues, which induces cross-organ sensitization through viscerovisceral and viscerasomatic convergence [23, 24]. Cortical output in response to pain activates descending pain regulatory circuitry in the brain stem and leads to the release of neurotransmitters in the dorsal horn of the spinal cord to regulate pain.

## 3. The Pathway of miRNA Maturation

miRNAs are small, single-stranded RNAs of about 22 nucleotides (nt). miRNAs can inhibit the expression of target genes to regulate protein expression at the post-transcriptional level [25–27]. The mechanism of this inhibition is that miRNAs complementarily bind with target mRNAs at the 3' untranslated regions (3'UTR) to block or suppress translation [28]. The miRNA biogenesis is complex and each step of the process is precisely regulated [29].

**Figure 1:**
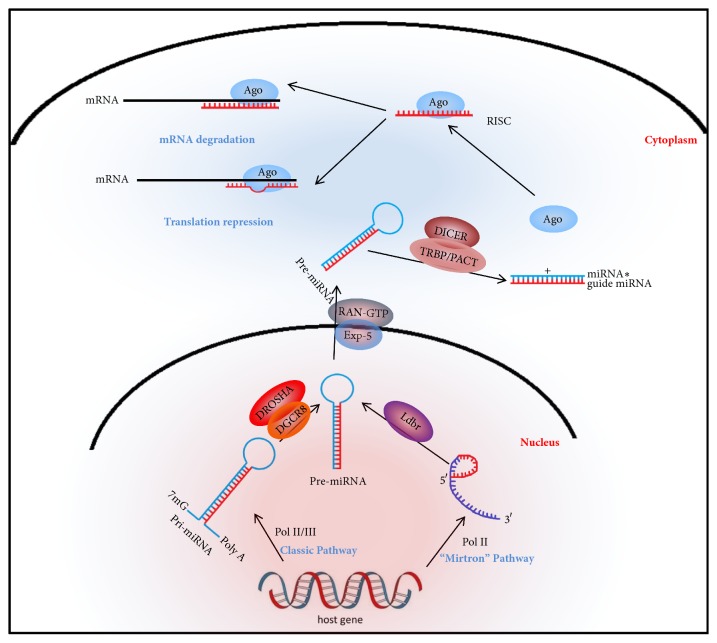
The classic pathway of miRNA biogenesis.

The classic pathway of miRNA biogenesis is generally known and shows a stepwise maturation pattern from the nucleus to the cytoplasm (Figure 1). miRNAs are thought to be transcribed from DNA that is not translated encompassing introns of coding genes, noncoding genes, and intergenic regions of genome [30–32]. But some miRNAs can also be encoded on exons since they have an overlap with transcription units [30, 33]. The classic pathway of miRNA maturation in mammalian cells can be generalized into 5 steps. First, the primary miRNA (pri-miRNA) is transcribed from host DNA by Type-II or Type-III RNA polymerases, which add a polyglandular (Poly A) tail at the 3' end, a 7-methylguanosine (7 mG) cap at the 5' end and a characteristic stem-loop structure [31, 34–37]. Second, the pri-miRNA transcript is processed in the nucleus by Drosha (a nuclear RNase) and DGCR8 (DiGeorge syndrome critical region 8, a RNA-binding protein) to a precursor miRNA (pre-miRNA) [28, 38–40]. The specific mechanism of this step is that the Drosha microprocessor complex recognizes the base of the stem-loop structure of the pri-miRNA and cleaves it to form a double-stranded pre-miRNA, which is about 70–90 nt long [28]. Third, the pre-miRNA is exported from the nucleus to the cytoplasm by exportin-5 (Exp-5, Ran-binding protein 21) and Ras-related nuclear protein (Ran) [41, 42]. Ran changes into the Ran-guanine triphosphatase (Ran-GTP) state and then the pre-miRNA/Exp-5/Ran-GTP complex is translocated to the cytoplasm through a nuclear pore complex [43]. Fourth, once in the cytoplasm, the RNase III enzyme Dicer, together with the TAR RNA-binding protein (TRBP) and protein activator of PKR (PACT) cleave the pre-miRNA into a 22 nt long imperfect double-stranded RNA with a 2 nt overhang at each of its 3' ends by removing the hairpin loop of the pre-miRNA [44]. One of the two strands is the mature miRNA (the guide strand) and the other one is the passenger strand (miRNA*∗*) [28, 36, 43]. Fifth, the mature miRNA combines with Argonaute (Ago) family proteins through the 2 nt 3' overhang to form the functional center of an RNA-induced silencing complex (RISC), which executes post-transcriptional gene silencing [45, 46]. If the 5' region of the miRNA (2-8 nt) can completely complement with its target mRNA, the target mRNA degrades and the translation process stops [29, 45, 47, 48]. At other times, imperfect base pairing between the 5' regions of the miRNA and its target mRNA leads to translation repression, which occurs mostly in animals [49]. Because of the partial complementarity between miRNAs and mRNAs, one miRNA may act on several target mRNAs and several miRNAs may target one mRNA. The miRNA*∗* does not change into a RISC complex and is discarded [50]. Occasionally, miRNAs can also bind to the 5' UTRs [51, 52] and even coding regions [53–55] of target mRNAs. Most miRNAs reduce protein expression, but some miRNAs can enhance translation such as miR369-3 and miR-373, which suggests complex mechanisms of miRNAs involved in gene regulation [56, 57].

Besides the classic pathway of miRNA biogenesis, a growing number of alternative miRNA pathways have been identified [58, 59]. The most important alternative miRNA maturation pathway is the “mirtron” pathway which does not depend on pri-miRNA Drosha/DGCR8 processing. The mirtron is spliced out from an inframe intron with a transient lariat shape that the 3' branch point is ligated to the 5' end of the intron. The lariat debranching enzyme (Ldbr) processes the lariat-shape intron into the pre-miRNA shape and then the pre-miRNA is transferred to the cytoplasm by Exp-5 [58]. Mirtrons share the same extra nuclear maturation process as the classic pathway of miRNAs.

## 4. The Role of miRNA in Visceral Pain

Since miRNAs can regulate target genes and proteins posttranscriptionally, it may participate in visceral pain processing through regulating neurotransmitters and their receptors as well as other proteins peripherally or centrally. To our knowledge, the role of miRNAs in visceral pain was first studied in BPS, which showed a correlation between the miRNAs miR-449b, miR-500, miR-328, and miR-320 and neurokinin 1 (NK1) receptor expression in BPS patients [70]. After that, many other miRNAs were reported as regulators in different animal models of visceral pain and human visceral pain disorders, including endometriosis, BPS/IC, IBS, and acute chest pain (Table 1).

Recent progress in understanding interventional functions of miRNAs facilitates examination of underlying mechanisms and translational potential. Technologies allowing accurate deletion of significant enzymes of miRNA synthesis or inhibition of specific miRNAs are getting more mature. Conditional deletion of Dicer in the dorsal root ganglion by using Nav1.8-Cre mice leads to the attenuation of nociception-related gene expression and the reduction of inflammatory pain while maintaining intact acute nociception [76]. Inhibition of specific miRNAs by administrating miRNA sponge lentivirus with inhibitor sequences provides a novel method to study the mechanisms of single miRNA accurately [60, 75].

### 4.1. miRNAs in Colonic Pain

IBS is a common gastrointestinal disorder characterized by chronic colonic pain. IBS afflicts 10-20% of the world population and is widely studied by gastroenterologists and researchers for its underlying mechanisms [77–79]. The etiology of IBS has been investigated ranging from early life stress, psychological disorders, and environmental effects to genetic factors [80–83]. IBS can be divided into three types by its changes in bowel habits, including chronic abdominal pain with frequent occurrence of diarrhea (IBS-D), constipation (IBS-C), or mixed bowel habits (IBS-M)[84].

A clinical study in IBS-D patients has emphasized the importance of miR-199 in visceral pain [60]. Specifically, gut miR-199a/b expression in IBS-D patients was significantly decreased, which was correlated directly with both increased visceral pain scores and TRPV1 expression. In a rodent model, intraperitoneal administration of lenti-miR-199a precursors upregulated miR-199a and decreased visceral hypersensitivity by attenuating TRPV1 signaling. Thus miR-199 precursors may be a promising therapeutic candidate for the treatment of IBS-D [60]. In another study, miR-24 was upregulated in intestinal mucosa epithelial cells of both IBS-D patients and a mouse model of trinitro-benzene-sulfonic acid induced IBS [61]. The authors identified that the serotonin reuptake transporter (SERT), which transports 5-hydroxytryptamine (5-HT, serotonin) from synaptic spaces into presynaptic neurons and removes 5-HT from the interstitial space, was a potential target gene of miR-24 through a luciferase reporter assay [61, 85, 86]. In a chronic stress induced-visceral pain model, there was a significant increase in the interleukin-6 (IL-6) signal transducer glycoprotein 130 (gp130) and miR-17-5p expression in the spinal cord while the signal transducer and activator of transcription 3 (STAT3) as well as glial fibrillary acidic protein (GFAP) expression significantly decreased [62]. STAT3 and gp130 are predicted to be targets of miR-17-5p and gp130 was upregulated with the increase of miR-17-5p. It is known that stress is able to downregulate spinal GFAP which is associated with changes in the expression of several molecules related to glutamatergic signaling, glutamine synthase, and proinflammatory cytokines [87]. The study suggests that there is a possible link between increased expression of miR-17-5p and activation of gp130/STAT3/GFAP, leading to neuroinflammation in visceral hypersensitivity conditions [62]. Another study identified the role of miR-150 and miR-342-3p in IBS [63]. Though the downstream proteins still remain to be determined, these two miRNAs are thought to be dysregulated in IBS and linked to pain and inflammatory processes [63]. However, in blood samples from endometriosis patients, the expression of miR-199 increases and miR-17-5p decreases compared to healthy controls, suggesting contradictory results to those in IBS studies [88]. It remains to be determined if alterations of specific miRNAs in the circulatory system, nervous system, and pathological tissues perform different functions.

Some studies reported that miRNAs contribute to intestinal hyperpermeability of IBS-D patients. In a clinical study, it was reported that in 42% of IBS patients, intestinal membrane permeability increased, which was correlated with an increase of miR-29a in the small bowel, colon tissues and blood microvesicles [64]. Moreover, the study identified that miR-29a interacted with complementary binding sites at the 3'UTR of the glutamate ammonia ligase gene, which led to the decrease of glutamine synthase [64]. Colonic glutamine helps maintain the intestinal barrier and reduce bacterial translocation. Thus, downregulation of glutamine synthase leads to increased intestinal permeability and chronic colonic hypersensitivity. In a study based on a 4% acetic acid-induced IBS-D rodent model, miR-144 was markedly upregulated and resulted in the downregulation of its target genes, occludin and zonula occludens 1 (ZO1), and two tight junction proteins, which regulate the colonic intracellular and paracellular permeability, respectively [65, 89, 90]. Under pathological conditions, specific miRNAs target genes contribute to intestinal epithelial barrier function and lead to visceral hypersensitivity through modulating intestinal permeability. Thus, miRNAs may be the potential diagnostic and therapeutic targets for IBS.

### 4.2. miRNAs in Pelvic Pain

miRNAs have been widely studied in pelvic pain. Endometriosis affects 5%–10% of premenopausal women worldwide but is not well managed [91, 92]. It is pathologically characterized by the presence of endometrial glands and stroma implanted outside the uterine cavity and may be asymptomatic or present with a wide range of symptoms including infertility, dysmenorrhea, pelvic pain, pelvic mass, and cancerous lesions [93–95]. However, the clinical diagnosis and pain management of endometriosis remain difficult due to the lack of understanding of underlying mechanisms [96].

A genome-wide association study (GWAS) on women with dysmenorrhea pain has identified one GWAS association at 1q13.2 that colocalizes with nerve growth factor (NGF), a neurotrophin linked to pain pathophysiology. This indicates that female pelvic pain severity is partly genetically determined [97]. Recently, the epigenetic mechanisms involved in endometriosis have also been widely studied. An early clinical study showed that miR-9 and miR-34 families were downregulated in endometrial tissues of women with painful endometriosis, but the downstream genes of the miRNAs regulation were not determined [66]. In a subsequent* in vitro* endometrial stroma cell study, an increase of miR-142-3p induced a significant decrease of steroid sulfatase and gp130 as well as deactivation of the IL-6-mediated inflammatory STAT3-pathway, resulting in decreased cell viability [67]. A recent study found that oxidized-lipoprotein (ox-LDL) levels in peritoneal fluid were related to maintenance of endometriotic lesions and nociception [68]. Specifically, the ox-LDL treatment of human endometrial cell-lines caused significant overexpression of nociceptive and inflammatory genes including NGF, IL-6, and prostaglandin E synthase 3 (PTGES3), as well as differential expression of 20 miRNAs including isoforms of miR-29, miR-181, and let-7 compared to control. These results were similar to the endometriotic tissues from endometriosis patients with pain compared to those without pain [68]. A prospective cohort study on women with endometriosis and those with pelvic pain but without the diagnosis of endometriosis showed that the serum and peritoneal fluid levels of IL-6, miR-122, and miR-199a were significantly higher in the former. In this study, there was no evidence suggesting the association between the expression of miR-122, miR-199a, and the severity of pelvic pain, but it provided evidence that miRNAs might serve as biomarkers for discrimination of endometriosis and other types of pelvic pain [69]. A clinical study of another intractable pelvic pain, vestibulodynia (VBD), showed patients with comorbid VBD and IBS failed to exhibit a balance in pro- and anti-inflammatory cytokines, while VBD patients compensated by increasing anti-inflammatory cytokines [98]. Both of these patients differentially expressed several miRNAs in peripheral blood which were predicted to be important for pain. All these studies of miRNAs on painful endometriosis are weighted in inflammatory-related pathways which might be a significant cause of pain in endometriosis and miRNAs could be promising analgesia targets. The studies support pain-related miRNA alterations in local pathological endometrial tissues or circulating biomarkers, but whether miRNAs at the spinal level or supraspinal level participate in endometriosis is still unclear.

miRNA activity has also been reported in another type of pelvic pain, bladder pain. BPS, also known as IC, is a syndrome characterized by pelvic pain related to urinary urgency and urinary frequency with multiple etiologies attributed to a recurrent and chronic inflammatory status of the muscle and submucosa of the bladder [99]. The specific etiology of BPS is not fully understood though pathogenic mechanisms including neuroinflammatory, autoimmune, possibly infectious, or toxic agents have been hypothesized [100].

As mentioned above, the first study of miRNAs in BPS showed that long-time exposure of bladder smooth muscle cells to substance P decreased NK1 receptor mRNA expression and concomitantly increased miR-449b and miR-500 [70]. The authors also identified this phenomenon in BPS patients and found that miR-449b and miR-500 increased, which indicated that activation of specific miRNAs caused an attenuation of NK1 receptor synthesis in BPS [70]. Using laser microdissection, Monastyrskaya et al. emphasized a possible association between miR-199a-5p expression and urothelial permeability in BPS patients [71]. Specifically, upregulation of miR-199a-5p and its concomitant downregulation of target genes, including LIN7C, ARHGAP12, PALS1, RND1, and PVRL1, might impact the urothelial barrier to induce defects in urothelial integrity leading to chronic bladder pain [71]. Hyperpermeability might be a significant reason for pelvic pain of BPS similar to IBS. Epithelial mesenchymal transition (EMT) and fibrosis in the bladder wall might change the permeability of the bladder, partially contributing to bladder pain. Recently, two studies on IC in postmenopausal women identified the role of miRNAs in EMT and fibrosis in the development of IC [72, 73]. In IC bladder tissues from postmenopausal women, a decrease of miR-214 and an increase of its target mitofusin 2 (Mfn2) compared to the normal bladder tissues were demonstrated by immunohistochemistry. In order to mimic the environment of IC patients to understand the underlying mechanisms, the authors transfected adipose-derived mesenchymal stem cells extracted from ovariectomized rats with miR-214 inhibitors and found downregulation of N-cadherin, fibronectin, Twist1 (twist basic helix-loop-helix transcription factor 1), Snail, and vimentin and upregulation of Mfn2, E-cadherin, and ZO1, resulting in promoting EMT and fibrosis in the bladder wall [72]. Another study showed a decrease in miR-139-5p and an increase in its target lysophosphatidic acid receptor 4 (LPAR4) in IC bladder tissues. Tissue from the IC group also showed a significant increase in expression of phosphatidylinositol 3-kinase (PI3K), Akt, p-PI3K, p-Akt, N-cadherin, vimentin, transforming growth factor-*β*1 (TGF--*β*1), and connective tissue growth factor (CTGF) and a decrease in expression of E-cadherin compared to the control group. These findings suggest that downregulation of miR-139-5p may advance EMT and fibrosis of the bladder by targeting LPAR4 and its downstream PI3K/Akt signaling pathway in postmenopausal IC [73]. The findings of the role of miRNAs in EMT and fibrosis in IC including miR-214 and miR-139-5p provide a novel insight into IC treatment.

Early adverse life events may render an individual more vulnerable to suffer chronic visceral hypersensitivity because of a failure of adaptive or coping mechanisms which prepare the individual to endure subsequent traumas better. This is an area currently being explored by epigenetic research. A study based on a rodent model has identified the involvement of miRNA-mediated post-transcriptional regulation of the developing spinal *γ*-aminobutyric acid- (GABA-) ergic system in neonatal cystitis-induced chronic visceral pain in rats [74]. Specifically, intravesicular injection of zymosan into the rats' bladder during early postnatal days induced neonatal cystitis and these rats showed upregulation of mature miR-181a in the L6-S1 spinal dorsal horn in adults. Further study demonstrated multiple complementary binding sites in miR-181a for GABA_A_ receptor subunit GABA_A*α*-1_ gene and an increase in miR-181a downregulated GABA_A*α*-1_ receptor subunit gene and protein expression in the spinal cord [74]. Recently, this laboratory confirmed the role of the spinal GABAergic system in bladder pain using the same model. Zymosan treatment in neonates and adults induced a similar increase in expression of spinal miR-92b-3p and a subsequent decrease in expression of its two targets, potassium chloride cotransporter (KCC2) and vesicular GABA transporter (VGAT), contributing to bladder nociception [75]. The impairment of GABAergic inhibition plays a key role in the transition of acute to chronic pain and several GABA-associated components, including GABA_A_ receptor subunits, KCC2, GABA synthesizing enzymes, and VGAT, have been reported to be involved in this process [101–104]. miRNA-mediated post-transcriptional regulation of the spinal GABAergic system may contribute to the long-lasting pelvic pain of cystitis, which provides novel targets for treatment of intractable pelvic pain.

### 4.3. Circulating miRNA in Chest Pain

Coronary artery disease, a major cause of chest pain with high mortality and morbidity worldwide, is divided into two subtypes: stable coronary heart disease and acute coronary syndrome (ACS). ACS is the symptomatic, clinical presentation of coronary artery disease, approximately half resulting from acute myocardial infarction, which causes death or disability within the first hours [105, 106]. For this reason, it is essential to discriminate ACS from other types of chest pain. Previous studies were weighing in the roles of circulating miRNAs as biomarkers in diagnosis of ACS or acute myocardial infarction compared to traditional diagnostic indices such as clinical symptoms, ECG changes, and elevation of cardiac troponin [107, 108]. It was reported that expression of miR-208b, miR-499, miR-146, miR-106b/25 cluster, miR-21/590-5p family, miR-17/92a, miR-451, miR-132, miR-186, miR-122, miR-3149, miR-221-3p, miR-210, miR-941, and miR-3162-3p increased and expression of miR-150 and miR-145 decreased in peripheral blood of ACS patients [105, 109–119]. Though these findings provide diagnostic value of circulating miRNAs in ACS, the role of miRNAs in acute chest pain management is still unknown and further studies are needed.

## 5. Conclusions and Future Strategies

Current clinical treatment of visceral pain is unsatisfactory. Studies on alterations of miRNAs involved in different types of visceral pain have revealed that they may contribute to proinflammatory states, attenuation of epithelial barrier function and impairment of GABAergic system function, verifying the status of miRNAs as important modulators in development and maintenance of visceral pain. However, most studies focus on miRNA alterations in local pathological tissue biopsies rather than the nervous system. Different miRNAs are involved in different types of visceral pain and diseases, resulting in complex and possibly discrete mechanisms. Thus, further studies are needed to explore the mechanisms underlying miRNAs contribution to visceral pain conditions at the spinal and supraspinal levels and explore new and effective treatment targets for visceral pain. Additionally, pain processing is always complex and may involve multiple mechanisms, and the interactions between miRNAs and other epigenetic mechanisms such as DNA methylation and histone acetylation remain to be determined. How miRNAs change by environmental cues and how their changes contribute to visceral pain are still unclear. Whether there are upstream regulatory mechanisms of miRNA alterations is also unclear. Because one miRNA may act on several target genes, avoiding the side effects which arise from other targets of the therapeutic miRNA is important, yet it is hardly studied. In conclusion, the literatures directly implicating miRNAs in visceral pain are still limited. More advanced animal models for different types of visceral pain or other visceral pain-related diseases are needed to be studied. Currently, there are still challenges for visceral pain treatment. The significant role of miRNAs underlying this disorder is becoming increasingly recognized; therefore it is believed that miRNAs will be a promising treatment target for visceral pain management.

## Figures and Tables

**Table 1:** Involvement of miRNAs in visceral pain.

Diseases	miRNAs	Tissues	Targets	References
IBS	miR-199 ↓	Human colon; Rat colon/DRG	TRPV1 ↑	[60]
	miR-24 ↑	Human/mouse intestinal mucosa	SERT ↓	[61]
	miR-17-5p ↑	Lumbar spinal cord	STAT3↓; gp130↑	[62]
	miR-150 ↑ miR-342-3p ↑	Human whole blood	-	[63]
	miR-29a ↑	Human small bowel and colon; human blood macrovesicles	Glutamate ammonia ligase ↓	[64]
	miR-144 ↑	Rat distal colonic epithelial cells	Occludin ↓; ZO1 ↓	[65]

Endometriosis	miR-9 ↓; miR-34 ↓	Human endometrial tissues	-	[66]
	miR-142-3p ↑	Endometrial stroma cells	Steroid sulfatase ↓; gp130 ↓	[67]
	miR-29 ↑; miR-181 ↑; let-7 ↑	ox-LDL treated human endometrial cell-lines	NGF ↑; IL-6 ↑; PTGES3 ↑	[68]
	miR-122 ↑; miR-199a ↑	Serum; peritoneal fluid	-	[69]

BPS/IC	miR-449b ↑ miR-500 ↑	Bladder smooth muscle cells	NK1 receptor↓	[70]
	miR-199a-5p ↑	Bladder smooth muscle; Mature bladder urothelium; Primary urothelial culture	LIN7C ↓; ARHGAP12 ↓; PALS1 ↓; RND1↓; PVRL1 ↓	[71]
	miR-214 ↓	Postmenopausal women's bladder tissue; Ovariectomized rats' APMSCs	Mfn2 ↑;	[72]
	miR-139-5p ↓	Postmenopausal women's bladder tissue	LPAR4 ↑	[73]
	miR-181a ↑	Rat spinal cord	GABA_A_ ↓	[74]
	miR-92b-3p ↑	Rat spinal cord	KCC2↓; VGAT ↓	[75]

## Abbreviations

ACS: Acute coronary syndrome
Ago: Argonaute
DGCR8: DiGeorge syndrome critical region 8
EMT: Epithelial mesenchymal transition
Exp-5: Exportin-5
GABA: *γ*-aminobutyric acid
GFAP: Glial fibrillary acidic protein
gp130: Glycoprotein 130
5-HT: 5-hydroxytryptamine
IBS: Irritable bowel syndrome
IC/BPS: Interstitial cystitis/bladder pain syndrome
IL-6: Interleukin-6
KCC2: Potassium chloride cotransporter
Ldbr: Lariat debranching enzyme
Mfn2: Mitofusin 2
miRNA: MicroRNA
NGF: Nerve growth factor
NK1: Neurokinin 1
Nt: Nucleotides
ox-LDL: Oxidized-lipoproteins
PACT: Protein activator of double-stranded RNA-activated protein kinase
PI3K: Phosphatidylinositol 3-kinase
pre-miRNA: Precursor miRNA
pri-miRNA: Primary miRNA
PTGES3: Prostaglandin E synthase 3
SERT: Serotonin reuptake transporter
Ran: Ras-related nuclear protein
RISC: RNA-induced silencing complex
STAT3: Signal transducer and activator of transcription 3
TRBP: Transactivation response RNA-binding protein
UTR: Untranslated regions
VBD: Vestibulodynia
VGAT: Vesicular GABA transporter
ZO1: Zonula occludens 1.
